# The great gerbil (*Rhombomys opimus*) as a host for tick species in Gurbantunggut Desert

**DOI:** 10.1186/s13071-024-06160-5

**Published:** 2024-02-07

**Authors:** Gang Liu, Wenbo Tan, Huiqian Wang, Xiaoshuang Han, Sándor Hornok, Shanshan Zhao, Ligu Mi, Suwen Wang, Meihua Yang, Yuanzhi Wang

**Affiliations:** 1 Key Laboratory for Prevention and Control of Emerging Infectious Diseases and Public Health Security, The XPCC, School of Medicine, Shihezi University, Shihezi, Xinjiang Uygur Autonomous Region People’s Republic of China; 2 Department of Parasitology and Zoology, University of Veterinary Medicine, Budapest, Hungary; 3 HUN-REN-UVMB Climate Change: New Blood-Sucking Parasites and Vector-Borne Pathogens Research Group, Budapest, Hungary; 4 Department of Forest, College of Agriculture, Shihezi University, Shihezi, Xinjiang Uygur Autonomous Region People’s Republic of China

**Keywords:** Gurbantunggut desert, Great gerbil, Ticks, Wild rodents

## Abstract

**Background:**

Rodents play an important role in the life cycle of ixodid and argasid ticks, particularly as hosts of larvae and nymphs. The great gerbil (*Rhombomys opimus*), the preferred prey item of several carnivores (e.g. the red fox and marbled polecat), is the dominant rodent species in the Gurbantunggut Desert in northwestern China. The aim of this study was to investigate tick species associated with different hosts in the habitat of great gerbils, including wildlife and livestock.

**Methods:**

During 2018–2023, ticks were removed from 326 great gerbils, two red foxes (*Vulpes vulpes*), three marbled polecats (*Vormela peregusna*), 35 pastured sheep (*Ovis aries*), and one long-eared desert hedgehog (*Hemiechinus auritus*) in the Gurbantunggut Desert. Ticks were identified according to standard morphological keys. Then, they were further analyzed by molecular and phylogenic methods based on two mitochondrial markers, *16S rDNA* and cytochrome* c* oxidase subunit I (*COI*) genes.

**Results:**

A total of 889 ticks were collected, representing five species. These included *Hyalomma asiaticum* (*n* = 425: 24 larvae, 79 nymphs and 322 adults), *Rhipicephalus turanicus* (*n* = 153: 2 nymphs and 151 adults), *Haemaphysalis erinacei* (*n* = 298: 4 larvae, 7 nymphs and 287 adults), *Ixodes acuminatus* (*n* = 7: 4 nymphs and 3 adults) and *Ornithodoros tartakovskyi* (6 adults). Based on *COI* sequences, molecular and phylogenetic analyses showed that (i) *I*. *acuminatus* from great gerbils and marbled polecats clustered with *I*. *acuminatus* reported from Europe; (ii)* O. tartakovskyi* found in northwestern China belonged to an independent clade; (iii) *Hy*. *asiaticum*, *R*. *turanicus* and *Ha*. *erinacei* had 100% sequence identities to conspecific ticks sampled previously in China.

**Conclusions:**

The great gerbil is an important host for the developmental stages of *I*. *acuminatus*, *O*. *tartakovskyi*, *Ha*. *erinacei*, *Hy*. *asiaticum* and *R*. *turanicus*, thus supporting the life cycle of several tick species which, as adults, parasitize predators (red fox and marble polecat) as well as pastured sheep and hedgehogs in the Gurbantunggut Desert. *Ixodes acuminatus* and *O*. *tartakovskyi* were found for the first time on great gerbil and marbled polecat, respectively.

**Graphical Abstract:**

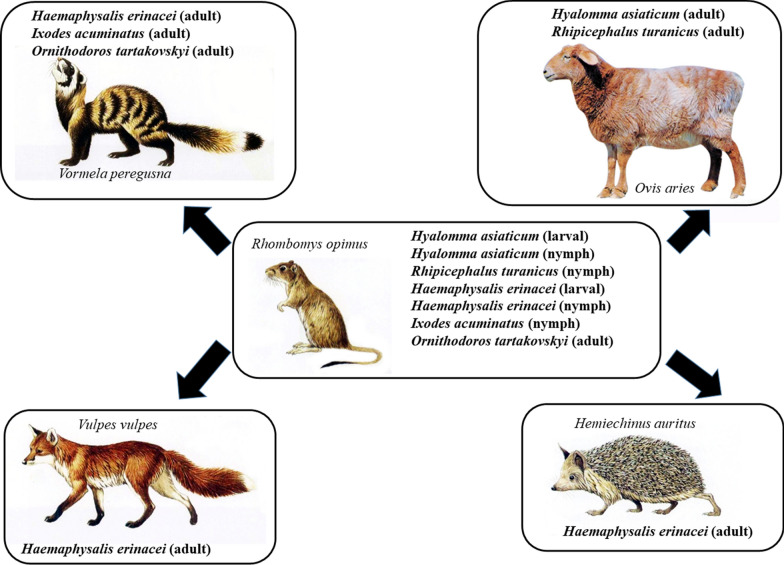

**Supplementary Information:**

The online version contains supplementary material available at 10.1186/s13071-024-06160-5.

## Background

**Fig. 1:**
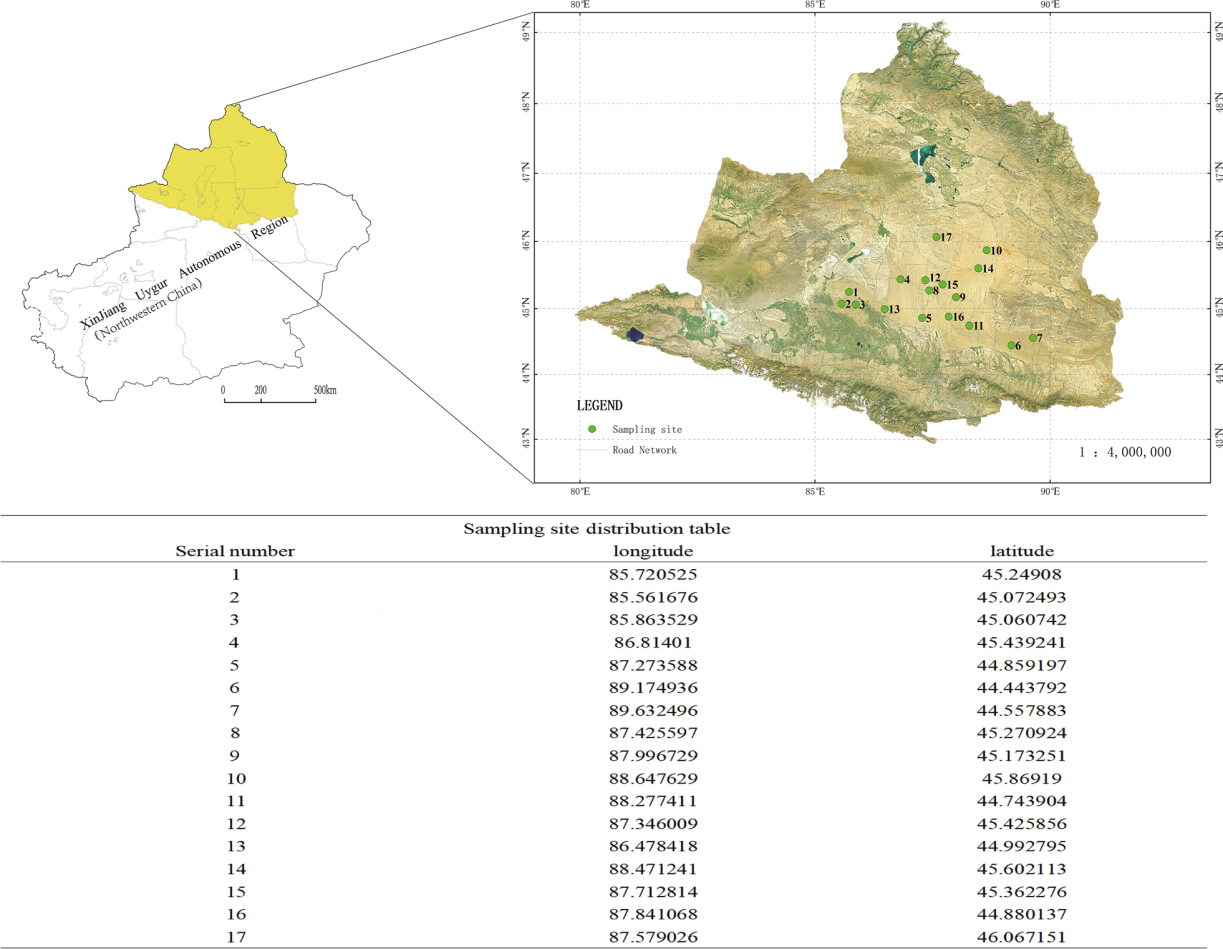
Sampling sites in Xinjiang Uygur Autonomous Region, northwestern China

The Gurbantunggut Desert, covering 4.88 × 10^4^ km^2^, is listed as the second largest desert in China. Its area ranges 84°50ʹ–91°20ʹE and 44°15ʹ–46°50ʹN [1] (Fig. 1). It lies in a typical temperate continental dry climate with annual precipitation of 70 to 150 mm. The annual average temperature ranges between 3 °C–7 °C, and its extreme high and low temperatures can reach > 40 °C and < − 40 °C, respectively. Its altitude ranges from 300 to 600 m a.s.l. The fixed and semi-fixed dunes account for 97% of the entire desert area. *Haloxylon ammodendron*, *Ha. persicum* and various ephemeral plants grow and provide food and water supplies for the great gerbils (*Rhombomys opimus*) [2, 3].

Meanwhile, > 100 animal species, including red fox (*Vulpes vulpes*), marble polecat (*Vormela peregusna*), long-eared hedgehog (*Hemiechinus auritus*) and goitered gazelle (*Gazella subgutturosa sairensis*) live in the area [4]. The great gerbil, the most dominant rodent species in the Gurbantunggut Desert, was previously reported to carry a variety of tick-borne pathogens, such as Tacheng tick virus 1, Songling virus [5], Karimabad virus [6], *Babesia* spp., *Theileria* spp. and *Anaplasma ovis* [7].

Ticks, such as *Hyalomma asiaticum*, *Rhipicephalus turanicus* and *Dermacentor marginatus*, were reported to be the most important pathogen vectors in the Gurbantunggut Desert [8]. To explore the relationship of local mammals as hosts of tick species shared between great gerbil and its predators or other sympatric mammals, this study aimed to systematically investigate ticks from great gerbils, marbled polecats (*V. peregusna*), a long-eared desert hedgehog (*H. auritus*), red foxes (*V. vulpes*) and pastured sheep (*O. aries*).

## Methods

### Sample collection and species identification

During 2018–2023, ticks were collected from 326 great gerbils (118 males, 208 females), three marbled polecats (road-killed), one long-eared desert hedgehog (road-killed), two red foxes (road-killed) and 35 pastured sheep (food supply for local oil workers) at 17 sampling sites in Gurbantunggut Desert (Fig. 1 and Additional file 1: Table S1). The great gerbils were captured in live traps according to our previous work [9] and were killed by cervical dislocation under the license of local Public Welfare Forest Reserve Management Station (PWFRMS). The road-killed wildlife were sent to our laboratory under the agreement of local PWFRMS. The pastured sheep were returned to local oil workers after sampling.

The presence of ticks was examined over the entire body of each animal at different intervals, including ears, neck, thorax, armpits, abdomen, interfeminium, crissum and so on. Ticks were collected with forceps and stored in 70% ethanol [7]. The ticks were morphologically observed using a stereo-microscope (Nikon SMZ-25, Japan) and identified to the species level according to the standard morphological keys as previously described [10].

### Sequencing and phylogenetic analysis of ticks

Genomic DNA was extracted from 1–15 ticks representing each tick species from each host species, using the TIANamp Genomic DNAKit (TIANGEN, Beijing, China). Morphological identification was confirmed by molecular and phylogenic analyses based on two mitochondrial markers, *16S rDNA* and cytochrome *c* oxidase subunit I (*COI*) genes [11]. In addition, phylogenetic analysis was performed using maximum likelihood method and 1000 bootstrap replicates with MEGA7.0.

### Statistical analysis

The numbers of tick-infested male and female great gerbils were compared according to sampling periods by the Fisher’s exact test (https://www.langsrud.com/fisher.htm). The difference was considered significant if *P* < 0.05.

## Results

### Tick identification

A total of 889 ticks were collected, including 425 *Hy*. *asiaticum* (24 larvae, 79 nymphs and 322 adults), 153 *R*. *turanicus* (2 nymphs and 151 adults), 298 *Haemaphysalis erinacei* (4 larvae, 7 nymphs and 287 adults), seven *Ixodes acuminatus* (4 nymphs and 3 adults) and six *Ornithodoros tartakovskyi* (all adults) (Table 1). Morphological characteristics of tick specimens are shown in Additional file 2: Fig. S1. Larvae and nymphs were only found on female great gerbils during the period between April and June, but ticks infested both female and male great gerbils during September and October. However, this difference was not significant (*P* = 0.057).

**Table 1:** Ixodid and argasid ticks collected from local wildlife and livestock in Gurbantunggut Desert

Animals (number)	Ticks					
	*Hyalomma asiaticum*	*Rhipicephalus turanicus*	*Haemaphysalis erinacei*	*Ixodes acuminatus*	*Ornithodoros tartakovskyi*	Total
*Rhombomys opimus* (326)	103 (24 larvae, 79 nymphs)	2 (nymphs)	11 (4 larvae, 7 nymphs)	4 (nymphs)	5 (adults)	125
*Ovis aries* (35)	322 (adults)	151 (adults)				473
*Vormela peregusna* (3)			273 (adults)	3 (adults)	1 (adults)	277
*Hemiechinus auritus* (1)			6 (adults)			6
*Vulpes vulpes* (2)			8 (adults)			8
Total	425	153	298	7	6	889

### Molecular and phylogenetic analyses

**Fig. 2:**
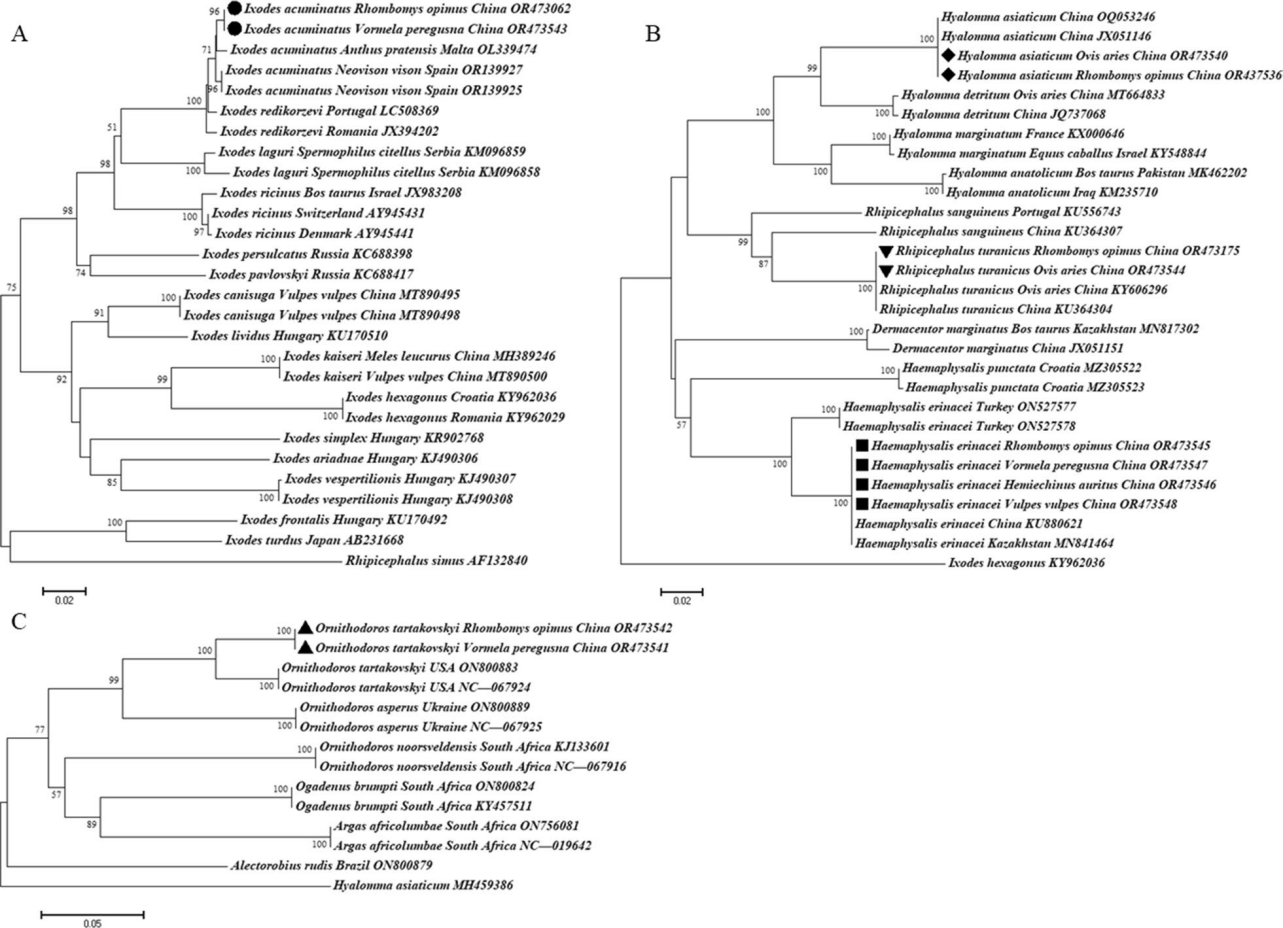
Maximum likelihood phylogenic tree inferred from the *COI* sequences of the ticks (**A*** Ixodes acuminatus*, **B** *Hyalomma asiaticum*, *Rhipicephalus turanicus* and *Haemaphysalis erinacei*, **C** *Ornithodoros tartakovskyi*) sampled from wildlife and pastured sheep in Gurbantunggut Desert, northwestern China. The new sequences provided by the present study are indicated by black circle/diamond/inverted triangle/square/triangle

A total of 71 sequences were included in the phylogenetic analysis, and 24 nucleotide sequences from this study had been deposited in the GenBank database (*16S rDNA*: OR474496.1, OR474529.1, OR475313.1, OR475314.1, OR475315.1, OR475316.1, OR476952.1, OR476978.1, OR478282.1, OR478283.1, OR478284.1, OR478285.1; *COI*: OR437536.1, OR473062.1, OR473175.1, OR473540.1, OR473542.1, OR473543.1, OR473544.1, OR473541.1, OR473546.1, OR473547.1, OR473545.1 and OR473548.1). Based on *COI* sequences, molecular and phylogenetic analyses showed that *I*. *acuminatus* (OR473062: from *R. opimus*, China, and OR473543: from *Vormela peregusna*, China) clustered with *I*. *acuminatus* (OR139927 and OR139925: from *Neovison vison*, Spain, and OL339474: from *Anthus pratensis*, Malta), and *Ixodes redikorzevi* (JX394202: unknown host, Romania, and LC508369: unknown host, Portugal) (Fig. 2A). *Hyalomma asiaticum*, *R*. *turanicus* and *Ha*. *erinacei* had 100% *COI* sequence identities to the corresponding tick species reported previously in China (Fig. 2B). *Ornithodoros tartakovskyi* from great gerbil and marbled polecat had identical *COI* sequences and formed a sister clade to those of a laboratory strain (ON800883 and NC_067924) (Fig. 2C).

## Discussion

This study provides, for the first time to our knowledge, the results of a large-scale sampling and molecular-phylogenetic analyses of ticks from wild rodents in the Gurbantunggut Desert, conducted in the frame of a 6-year-long survey.

Among the tick species identified, *I. acuminatus* is known to prefer rodents as hosts [10]. Based on morphological comparison of female specimens of *I*. *acuminatus* from Italy and France, and *I. redikorzevi* from the former USSR, *I*. *redikorzevi* was suggested to be a junior synonym of *I*. *acuminatus* [12], as also stated elsewhere [13]. Other literature data attest that both are valid species, until the opposite is proven by extensive morphological-molecular comparisons [14, 15]. In this study, the *COI* sequence of *I*. *acuminatus* (OR473062.1, OR473543.1) shared 98.73–99.39% identities with those of *I*. *acuminatus* from Spain and Malta and *I*. *redikorzevi* from Romania and Portugal. Interestingly, 100% identity was shown among *COI* amino acid sequences of ticks that were morphologically identified as either *I*. *acuminatus* or *I*. *redikorzevi* in distant parts of the Palearctic (Additional file 3: Fig. S2). These data support that *I. acuminatus* and *I. redikorzevi* might represent the same species. However, the ultimate conclusion could only be drawn in this context if detailed morphological analyses were possible between ticks that are near-identical in their barcoding gene and/or the corresponding amino acid sequences.

Based on literature data, *I*. *acuminatus* is widely distributed in the Palearctic region, including Belgium, France, Germany, UK, Italy, Hungary, Portugal, Spain, Malta and Turkey [10, 14, 16–19]. This tick species inhabits mainly temperate broad-leaf and mixed forests, where its main hosts are from families Erinaceidae, Mustelidae (including *Mustela*), Canidae, Felidae, Viverridae, Soricidae, Talpidae, Arvicolidae, Cricetidae (including *Microtus*), Gliridae, Muridae, Turdidae, Phasianidae, Accipitridae, Sylviidae and Troglodytidae [10, 14, 16–19]. At the same time, *I*. *redikorzevi* was reported to occur in the former USSR, Iran, Pakistan, Afghanistan, Nepal, Israel, Egypt, Kazakhstan, Turkey, Portugal and Romania, where it was collected from Corvidae, Turdidae, Paridae, Passeridae, Ploceidae, Mustelidae (including *Mustela*), Canidae, Sciuridae, Circetidae (including *Microtus*), Muridae, Talpidae and Erinaceidae [20–25]. Thus, finding seven *I*. *acuminatus* specimens in this study on great gerbil (*Rhombomys*, Circetidae) and marble polecat (*Vormela*, Mustelidae) in the Gurbantunggut Desert extends the above host range.

Previously, *Ha*. *erinacei* was collected from North African hedgehog (*Atelerix algirus*), European hedgehog (*Erinaceus europaeus*), white-breasted hedgehog (*Erinaceus concolor*), long-eared hedgehog (*Hemiechinus auritus*) and desert hedgehog (*Paraechinus aethiopicus*) in North Africa, Balkan peninsula, Italy, Pakistan, Afghanistan, Kazakhstan, Saudi Arabia, Turkey, Algeria, Bulgaria, Croatia, Jordan, Iran, Iraq, Israel, Kazakhstan, Kyrgyzstan and Uzbekistan [10]. In China, *Ha*. *erinacei* was reported to infest marbled polecat (*Vormela peregusna*) and red fox (*Vulpes vulpes*) [26–28]. In this study, 298 *Ha*. *erinacei* specimens were collected, including four larvae and seven nymphs from great gerbil, as well as 287 adults from marbled polecat, long-eared desert hedgehog and red fox. Based on these results, the marbled polecat was the dominant host of *Ha*. *erinacei*, especially in the adult stage of ticks. Interestingly, larvae and nymphs of *Ha*. *erinacei* infested great gerbils, which is known to be the most important prey item of marbled polecats [4]. This finding indicates that the co-habitation of *Ha*. *erinacei* with both the great gerbil and the marbled polecat is an integral aspect of the development of this tick species.

The geographical range of *O*. *tartakovskyi* in the Palearctic region covers Kazakhstan, Uzbekistan, Turkmenistan, Kyrgyzstan, Tajikistan, Iran, Czechia and China [29], and its natural hosts include great gerbils and Central Asian tortoise (*Testudo horsfieldii*) [29]. In this study, five ticks identified morphologically as *O*. *tartakovskyi* from great gerbils and marbled polecats shared 93.43% sequence identity to an *O*. *tartakovskyi* laboratory strain. Further compared at the level of amino acids, they had 100% identity (Additional file 4: Fig. S3). These findings add marbled polecats to the host range of *O*. *tartakovskyi*.

In addition, the immature stages of *Hy*. *asiaticum* and *R*. *turanicus* were found on great gerbils, and their adults on pastured sheep. This finding indicates that the great gerbil plays an important role in maintaining the larvae and nymphs of *Hy*. *asiaticum* and *R*. *turanicus*, while their adults use especially ungulates as reproductive hosts in the Gurbantunggut Desert. These results are consistent with our previous work [30].

Lastly, as our sampling sites were located in deep region of Gurbantunggut Desert rather than in the oasis of the Junggar Basin, some tick species, such as *Dermacentor marginatus* and *D. nuttalli* [30], were not found in this investigation. Moreover, the marbled polecats, red foxes and great gerbils served as reservoirs for spotted fever *Rickettsia*, *Babesia* and novel tick-borne bunyaviruses [5, 26–28]. In the future, there will be more work on ticks and tick-borne pathogens in the Gurbantunggut Desert.

In this study, tick larvae and nymphs were mostly found on female great gerbils, which might be related to the following: (i) the breeding population of great gerbils usually consists of an adult male and 1–3 females (sometimes up to 6–7 females), and the latter feed their young in burrows where nidicolous immature ticks stay [31]; (ii) female great gerbils need to eat *Haloxylon* and ephemeral plants to obtain more food and water supply in stages of fetation and nursing, especially during April–June [2, 3]. However, larvae and nymphs were found on both female and male great gerbils during September and October, which might be related to the availability of more sub-adult gerbils among sampled hosts.

## Conclusions

Based on the morphological and molecular-phylogenetic characters five tick species were identified, for which great gerbil acts as the primary host compared to the other desert animals including marbled polecat, long-eared desert hedgehog, red fox and pastured sheep (Additional file 5: Fig. S4). The indigenous status of *I*. *acuminatus* in China was confirmed by both morphological and molecular analyses. Two tick species were reported from their hosts for the first time, i.e. *I*. *acuminatus* from great gerbil and *O*. *tartakovskyi* from marbled polecat.

### Supplementary Information

**Additional file 1: Table S1.** Information on sampled hosts and ticks in Gurbantunggut Desert.

**Additional file 2: Figure S1.** Morphological characteristics of *Hyalomma asiaticum*, *Rhipicephalus turanicus*, *Ixodes acuminatum*, *Haemaphysalis erinacei* and *Ornithodoros tartakovskyi*.

**Additional file 3: Figure S2.** Nucleic acid (A) and amino acid (B) sequence comparison of *Ixodes acuminatus* and *Ixodes redikorzevi*.

**Additional file 4: Figure S3.** Nucleic acid (A) and amino acid (B) sequence comparison of *Ornithodoros tartakovskyi*.

**Additional file 5: Figure S4.** Schematic diagram illustrating the connectedness of tick hosts in the Gurbantunggut Desert.

## Data Availability

The sequences obtained and analyzed during the present study were deposited in the GenBank database (*16S rDNA*: OR474496.1, OR474529.1, OR475313.1, OR475314.1, OR475315.1, OR475316.1, OR476952.1, OR476978.1, OR478282.1, OR478283.1, OR478284.1, OR478285.1; *COI*: OR437536.1, OR473062.1, OR473175.1, OR473540.1, OR473542.1, OR473543.1, OR473544.1, OR473541.1, OR473546.1, OR473547.1, OR473545.1 and OR473548.1).

## Abbreviation

COI: Cytochrome *c* oxidase subunit I
