# Supplementary material for: The great gerbil (Rhombomys opimus) as a host for tick species in Gurbantunggut Desert
Source: Parasit Vectors. 2024 Feb 7;17:55. doi: 10.1186/s13071-024-06160-5 (PMC10851595; doi:10.1186/s13071-024-06160-5)
Supplement: Supplementary file 1 — Additional file 1: Table S1. Information on sampled hosts and ticks in Gurbantunggut Desert. [file 13071_2024_6160_MOESM1_ESM.docx]

**Table S1**. The information on sampled hosts and ticks in Gurbantunggut desert.

| Sampling site | Sampling time | Number of hosts | Age of hosts in years | Sex ratio of hosts (♂/♀) | Infestation rate of hosts | Number of male and female host infested by ticks (♂/♀) | Infestation rate of male and female hosts (♂/♀) | The number of ticks obtained | Ratio of not engorged ticks to engorged ticks (NT/ET) | Number of ticks (if adults: sex ratio ♂/♀) |
| --- | --- | --- | --- | --- | --- | --- | --- | --- | --- | --- |
| 1 | May 2018 | 14 (adult GG) | 1-3* | 3/11 | 2/14 (14.28%) | 0/2 | 0%/18.18% | 3 | 0/3 | 3 |
| 2 | April 2020 | 21 (adult GG) | 1-3* | 6/15 | 2/21 (9.52%) | 0/2 | 0%/13.33% | 2 | 0/2 | 2 |
|  |  | 1 (adult MP) | ~2 | 0/1 | 1/1 (100.00%) | 0/1 | 0%/100.00% | 111 | 91/20 | 35/76 |
|  |  | 35 (adult PS) | ~1 | 35/0 | 35/35 (100%) | 35/0 | 100.00%/0% | 473 | 162/311 | 72/401 |
| 3 | April 2023 | 13 (adult GG) | 1-3* | 3/10 | 0/13 (0%) | 0/0 | 0%/0% | 0 | 0 | 0 |
| 4 | October 2019 | 17 (adult GG) | 0.5-3* | 8/9 | 7/17 (41.17%) | 2/5 | 25.00%/55.56% | 15 | 0/15 | 15 |
| 5 | May 2022 | 10 (adult GG) | 1-3* | 2/8 | 0/10 (0%) | 0/0 | 0%/0% | 0 | 0 | 0 |
|  |  | 1 (adult DH) | ~2 | 0/1 | 1/1 (100.00%) | 0/1 | 0%/100.00% | 6 | 2/4 | 2/4 |
| 6 | June 2022 | 16 (adult GG) | 1-3* | 4/12 | 4/16 (25.00%) | 0/4 | 0%/33.33% | 6 | 1/5 | 6 |
| 7 | May 2023 | 18 (adult GG) | 1-3* | 5/13 | 1/18 (5.56%) | 0/1 | 0%/7.69% | 1 | 0/1 | 1 |
|  |  | 1 (adult MP) | ~3 | 0/1 | 1/1 (100.00%) | 0/1 | 0%/100.00% | 103 | 81/22 | 26/77 |
| 8 | May 2018 | 14 (adult GG) | 1-3* | 3/11 | 2/14 (14.29%) | 0/2 | 0%/18.18% | 3 | 0/3 | 3 |
| 9 | October 2021 | 27 (adult GG) | 0.5-3* | 13/14 | 7/27 (25.92%) | 1/6 | 7.69%/42.86% | 17 | 0/17 | 17 |
| 10 | April 2019 | 19 (adult GG) | 1-3* | 4/15 | 0/19 (0%) | 0/0 | 0%/0% | 0 | 0 | 0 |
|  |  | 1 (adult RF) | ~3 | 1/0 | 1/1 (100.00%) | 1/0 | 100.00%/0% | 5 | 5/0 | 0/5 |
| 11 | October 2021 | 29 (adult GG) | 0.5-3* | 15/14 | 8/29 (27.59%) | 2/6 | 13.33%/42.86% | 14 | 0/14 | 14 |
| 12 | April 2020 | 17 (adult GG) | 1-3* | 4/13 | 0/17 (0%) | 0/0 | 0%/0% | 0 | 0 | 0 |
| 13 | September 2020 | 25 (adult GG) | 0.5-3* | 11/14 | 5/25 (20.00%) | 1/4 | 9.09%/28.57% | 7 | 0/7 | 7 |
| 14 | October 2018 | 33 (adult GG) | 0.5-3* | 17/16 | 14/33 (42.42%) | 3/11 | 17.65%/68.75% | 26 | 1/25 | 26 |
| 15 | April 2021 | 8 (adult GG) | 1-3* | 2/6 | 0/8 (0%) | 0/0 | 0%/0% | 0 | 0 | 0 |
|  |  | 1 (adult MP) | ~2 | 0/1 | 1/1 (100.00%) | 0/1 | 0%/100.00% | 63 | 51/12 | 10/53 |
| 16 | October 2019 | 28 (adult GG) | 0.5-3* | 13/15 | 22/28 (78.57%) | 8/14 | 61.54%/93.33% | 31 | 2/29 | 31 |
| 17 | April 2022 | 17 (adult GG) | 1-3* | 5/12 | 0/17 (0%) | 0/0 | 0%/0% | 0 | 0 | 0 |
|  |  | 1 (adult RF) | ~2 | 0/1 | 1/1 (100.00%) | 0/1 | 0%/100% | 3 | 3/0 | 0/3 |
| total |  | 367 |  |  |  |  |  | 889 |  |  |

Abbreviations: GG=great gerbil; RF=red foxes; MP=marbled polecats; PS=pastured sheep; DH=long-eared desert hedgehog; ET=engorged tick; NT=not engorged tick.

Symbol: *(asterisk) means that >90% hosts belong to the provided age range.
