# Supplementary material for: The great gerbil (Rhombomys opimus) as a host for tick species in Gurbantunggut Desert
Source: Parasit Vectors. 2024 Feb 7;17:55. doi: 10.1186/s13071-024-06160-5 (PMC10851595; doi:10.1186/s13071-024-06160-5)
Supplement: Supplementary file 2 — Additional file 2: Figure S1. Morphological characteristics of Hyalomma asiaticum, Rhipicephalus turanicus, Ixodes acuminatum, Haemaphysalis erinacei and Ornithodoros tartakovskyi. [file 13071_2024_6160_MOESM2_ESM.pdf]

# Ticks from wildlife and pastured sheep in the Gurbantunggut desert

# Ticks from great gerbil (*Rhombomys opimus*)

Figure 1.

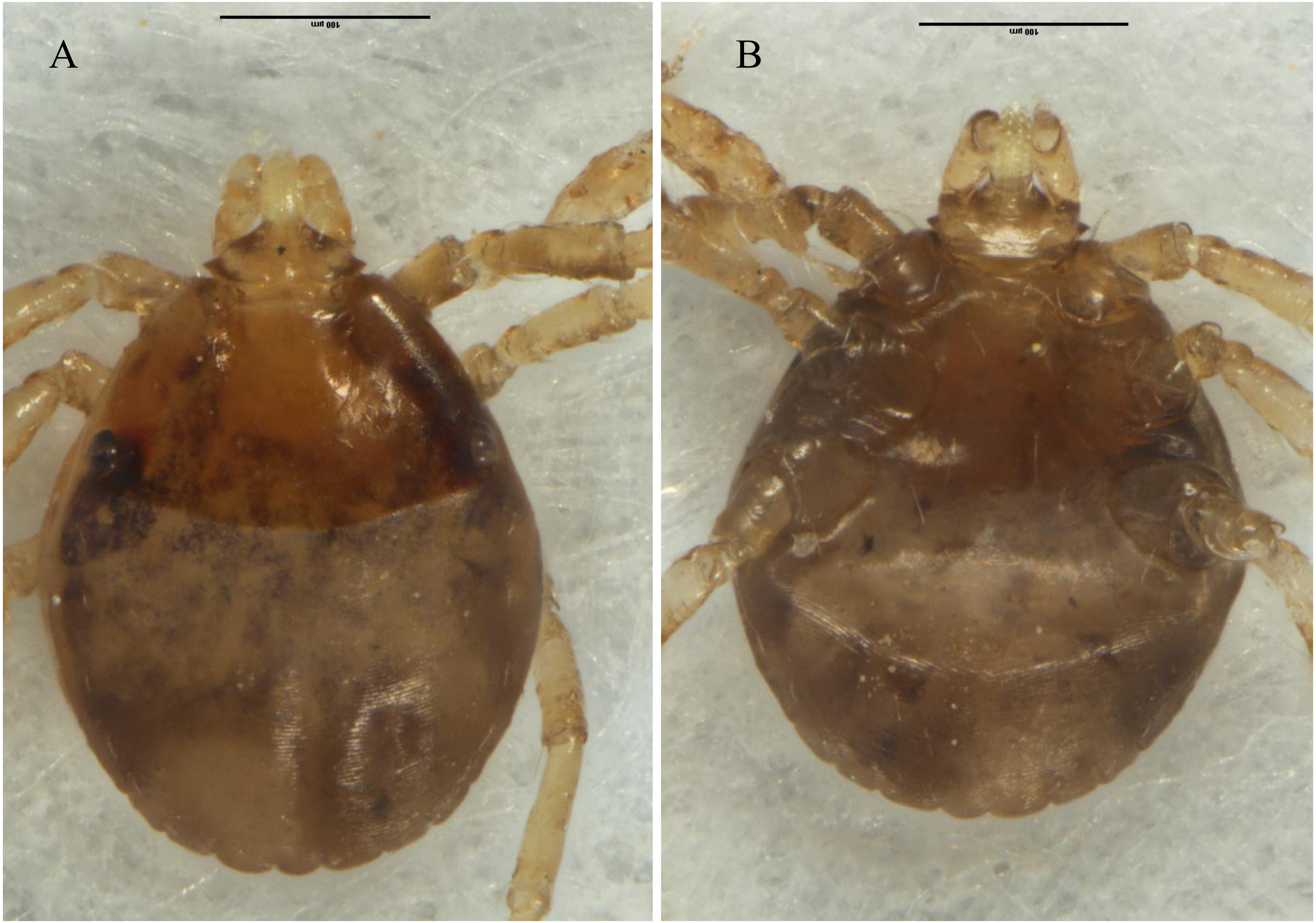

Figure 2.

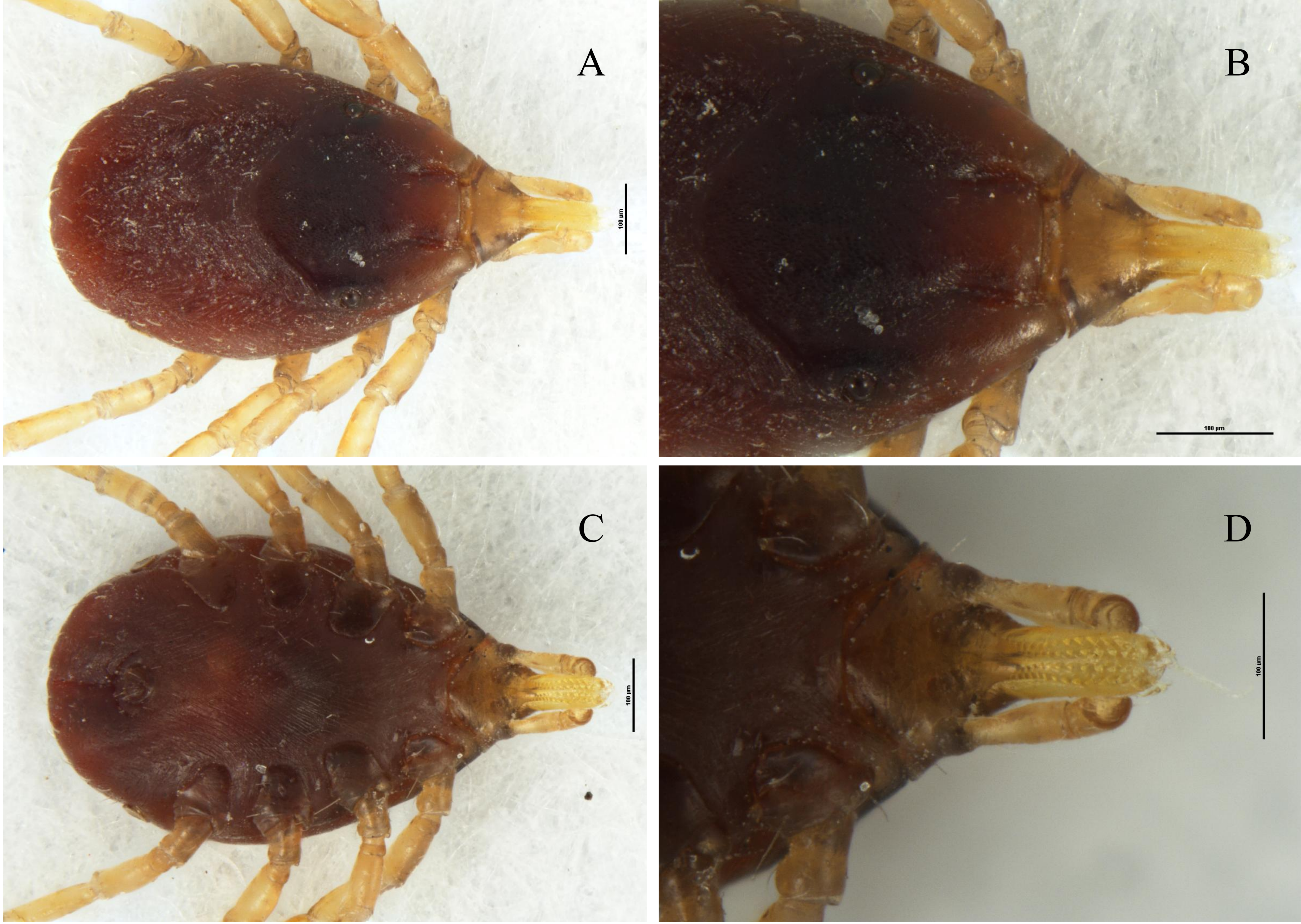

Figure 3.

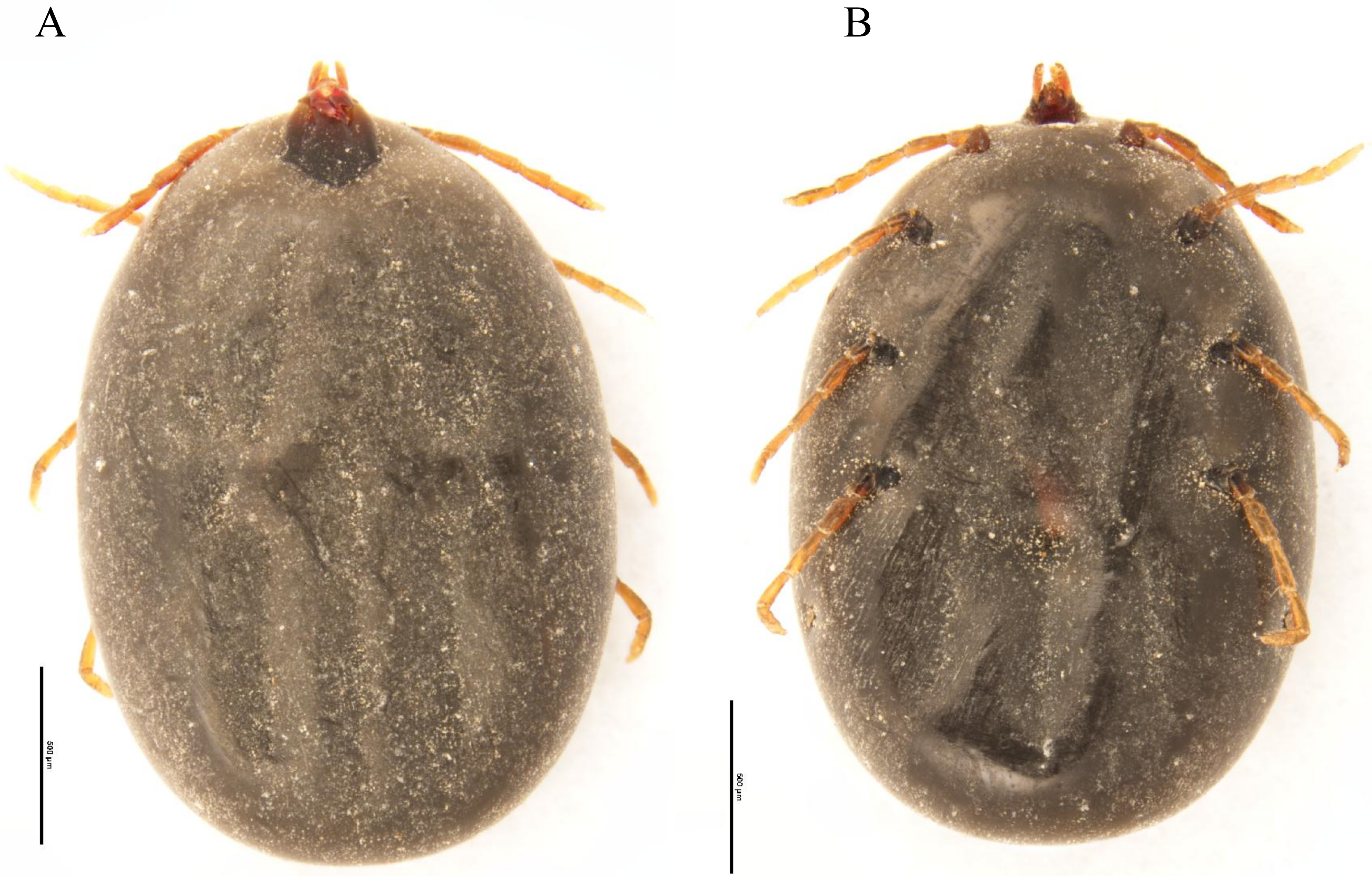

Figure 4.

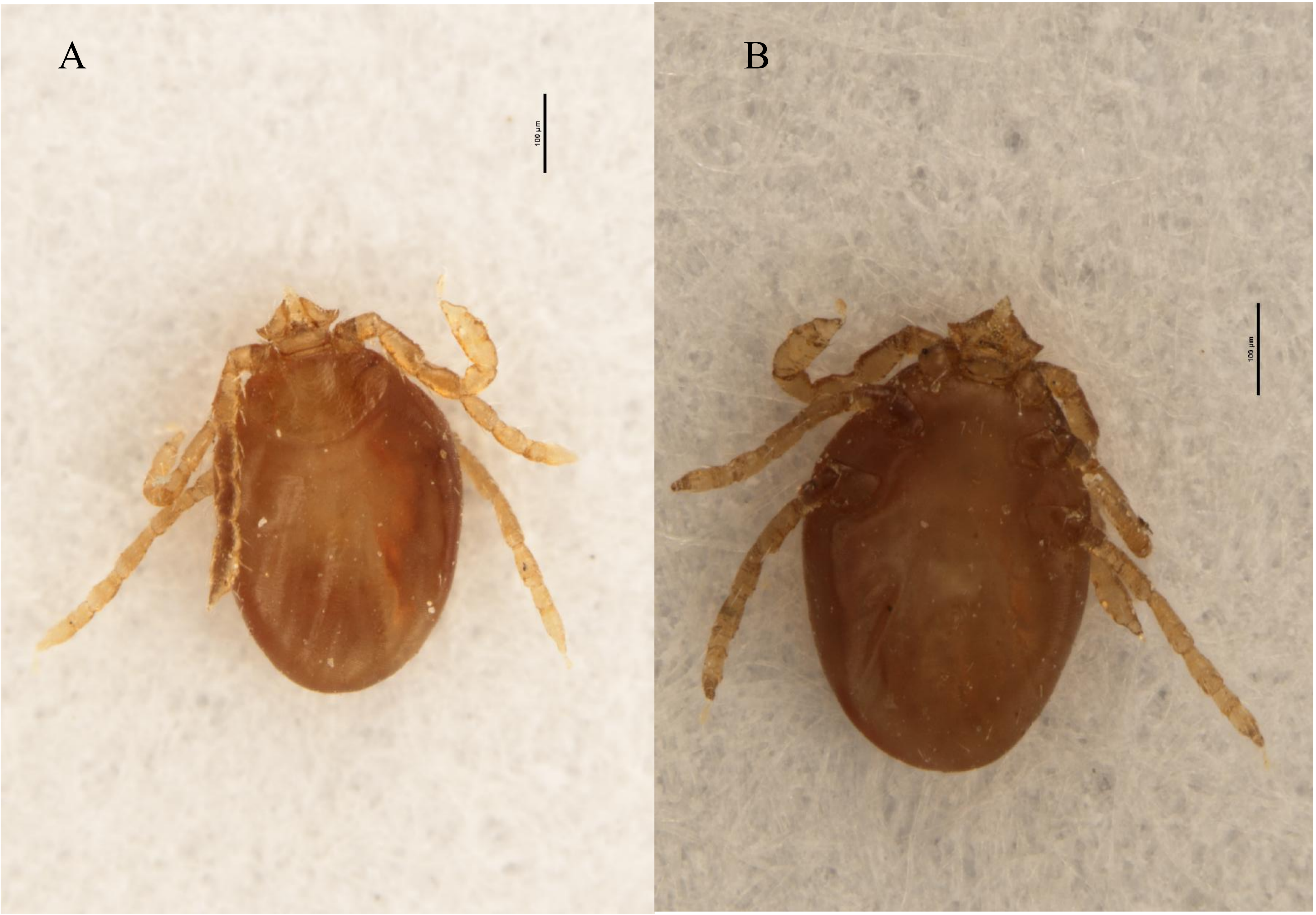

Figure 5.

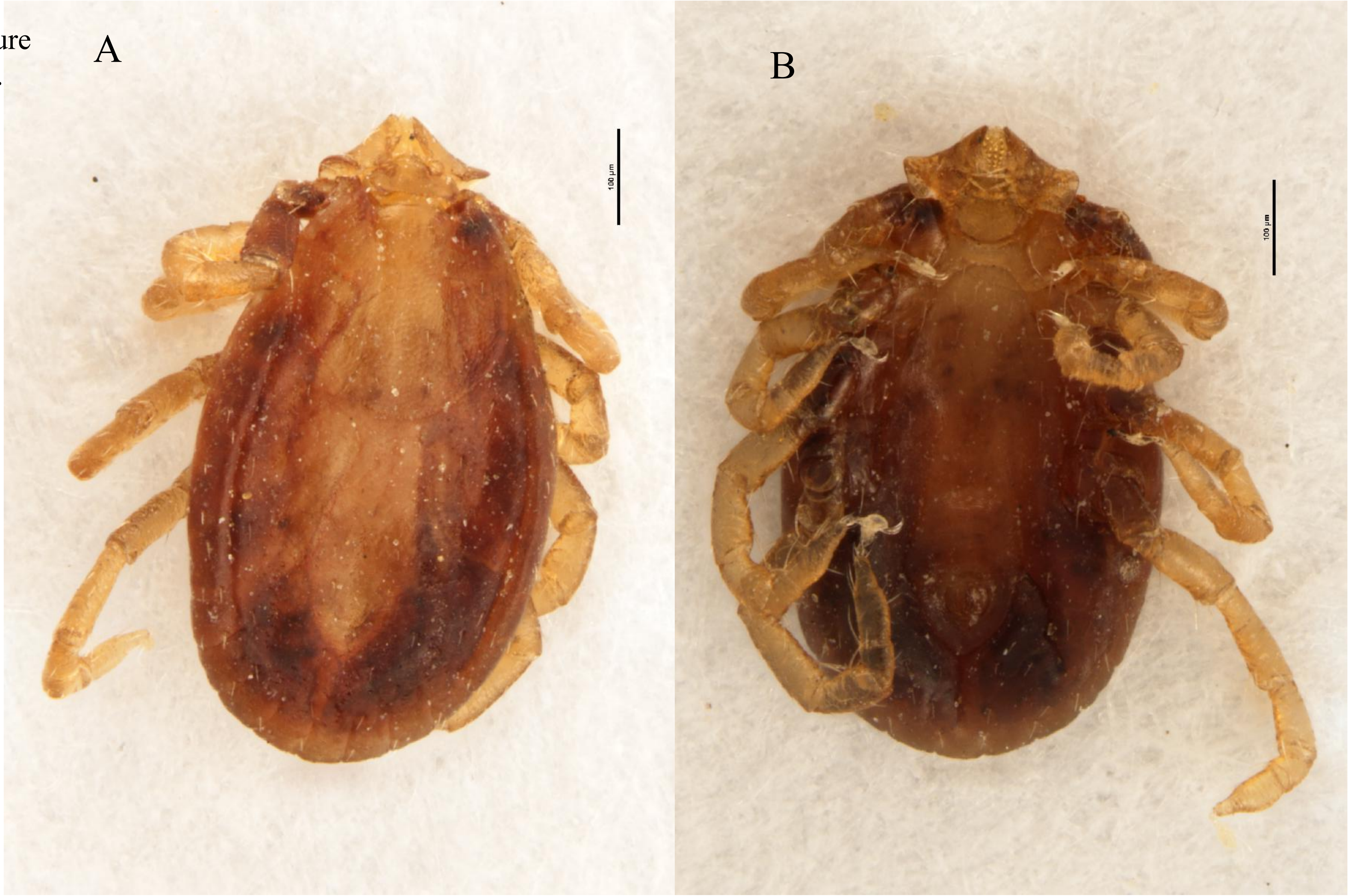

Figure 6.

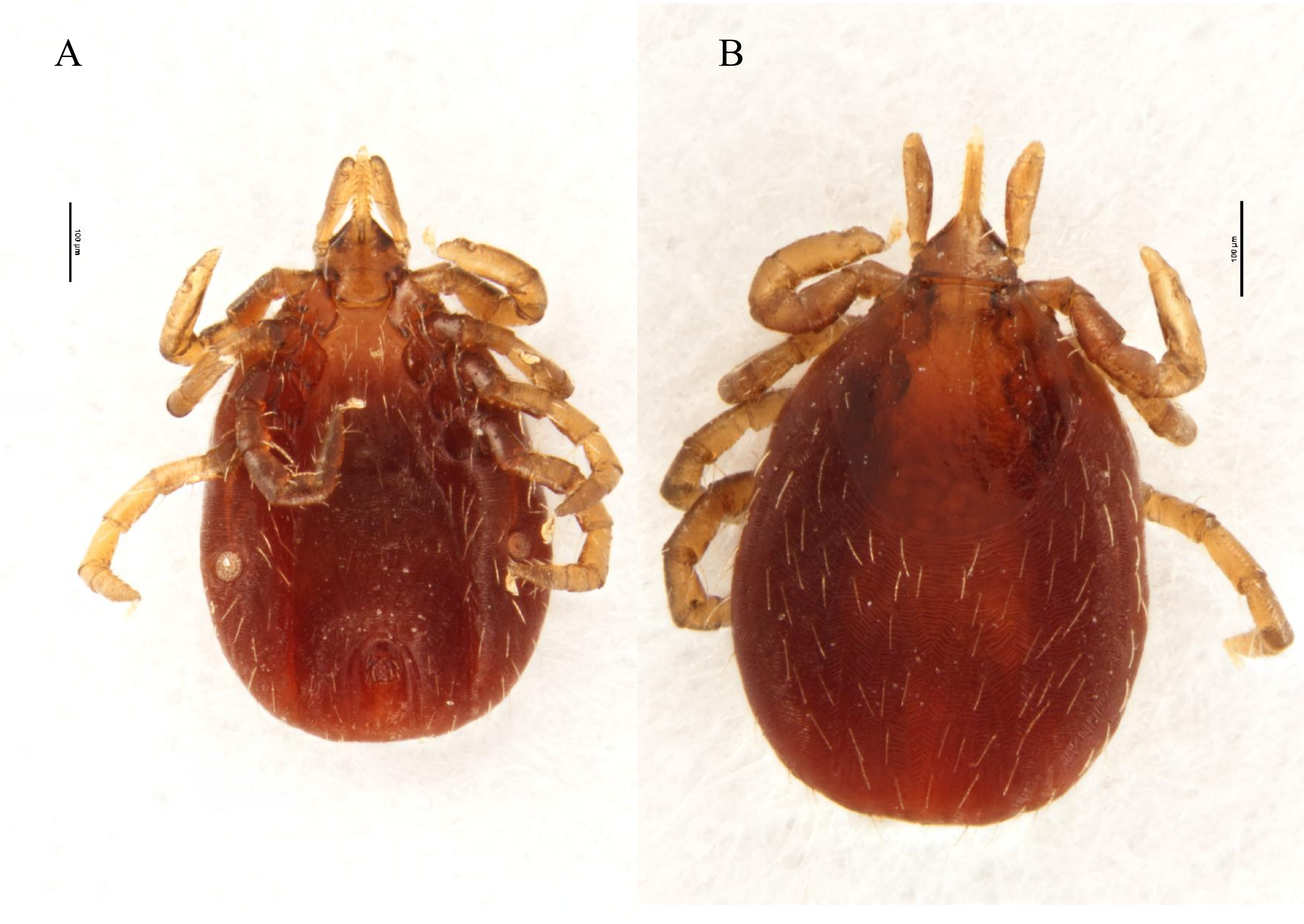

Figure 7.

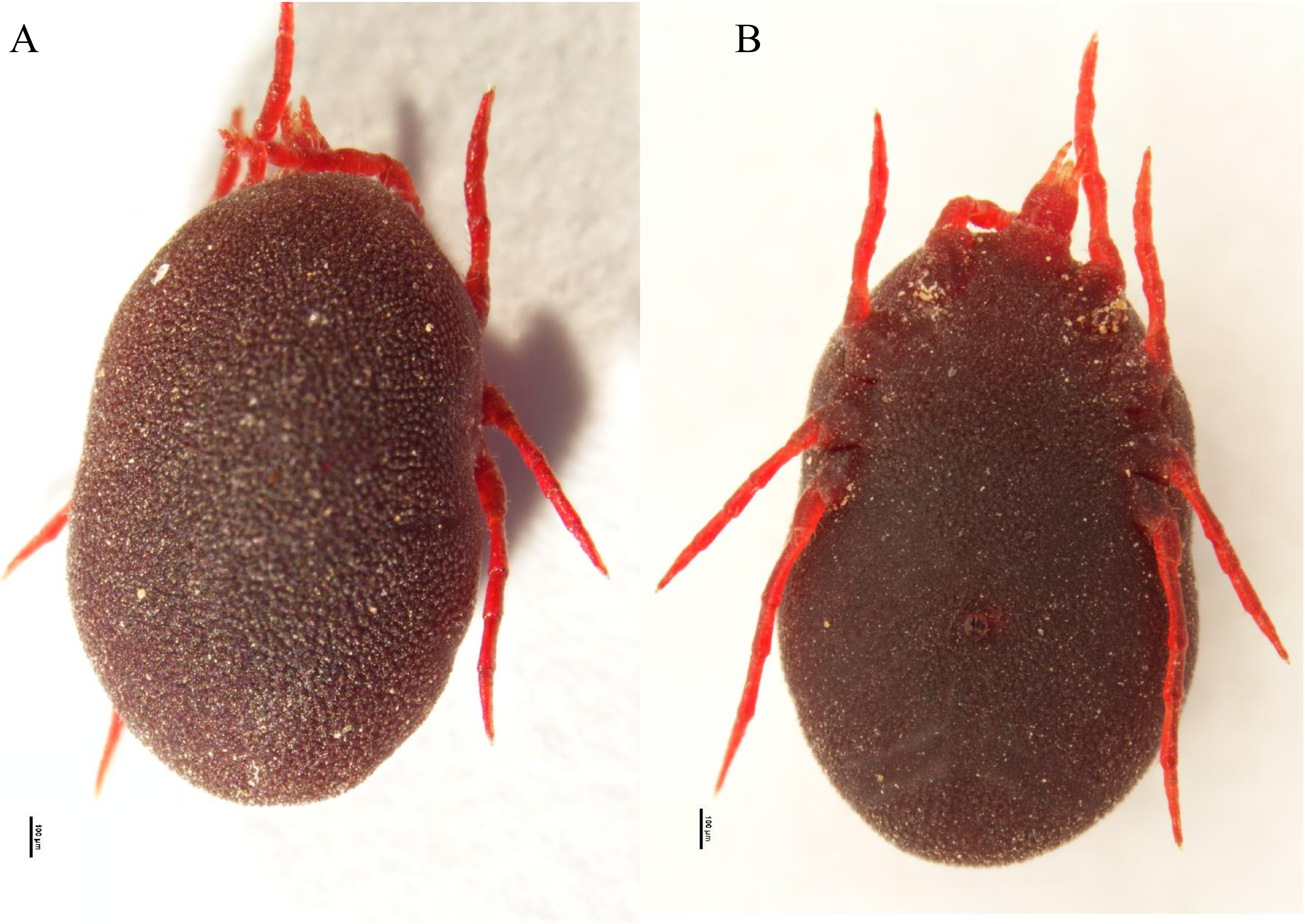

# Ticks from pastured sheep (*Ovis aries*)

Figure 8.

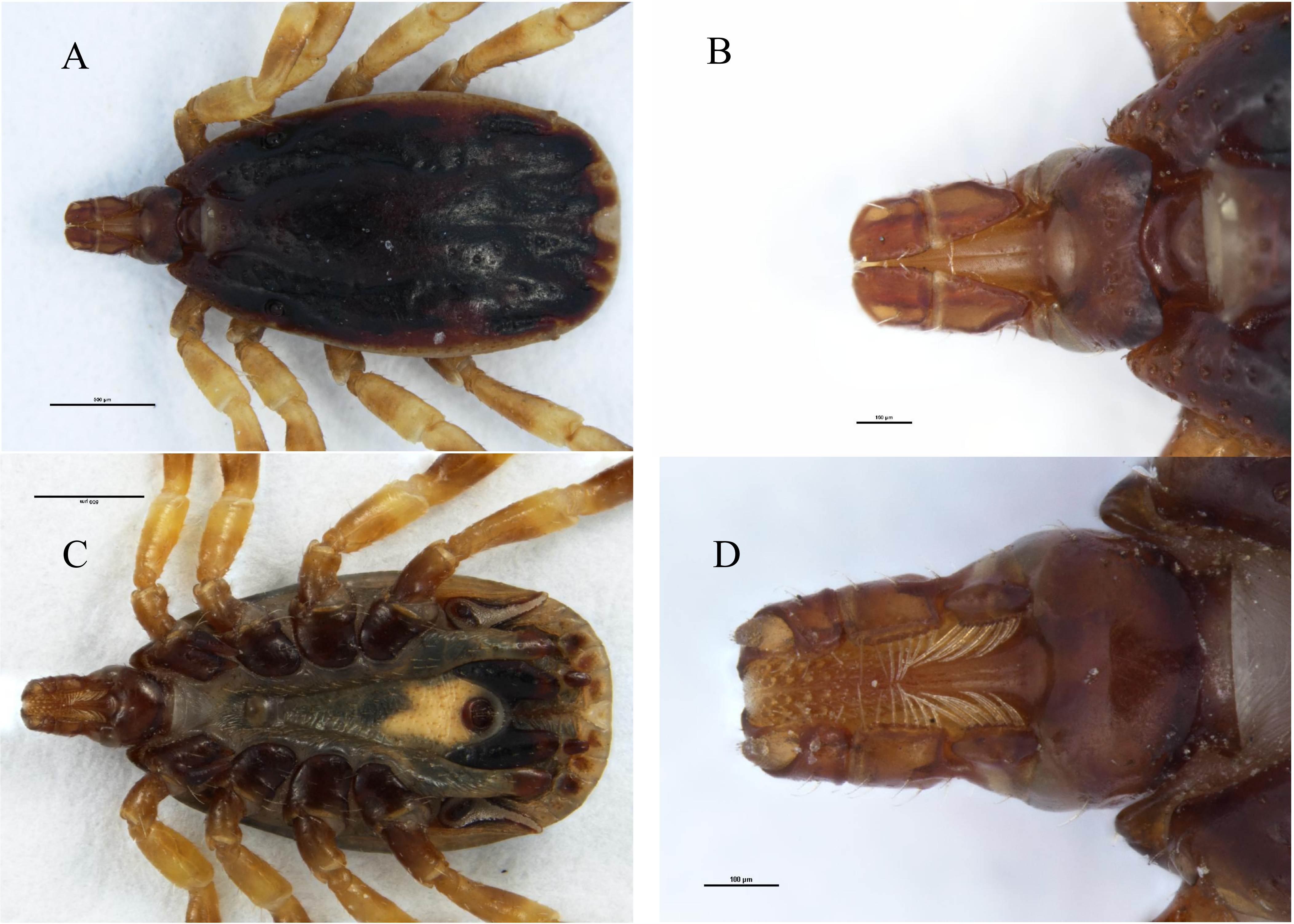

Figure 9.

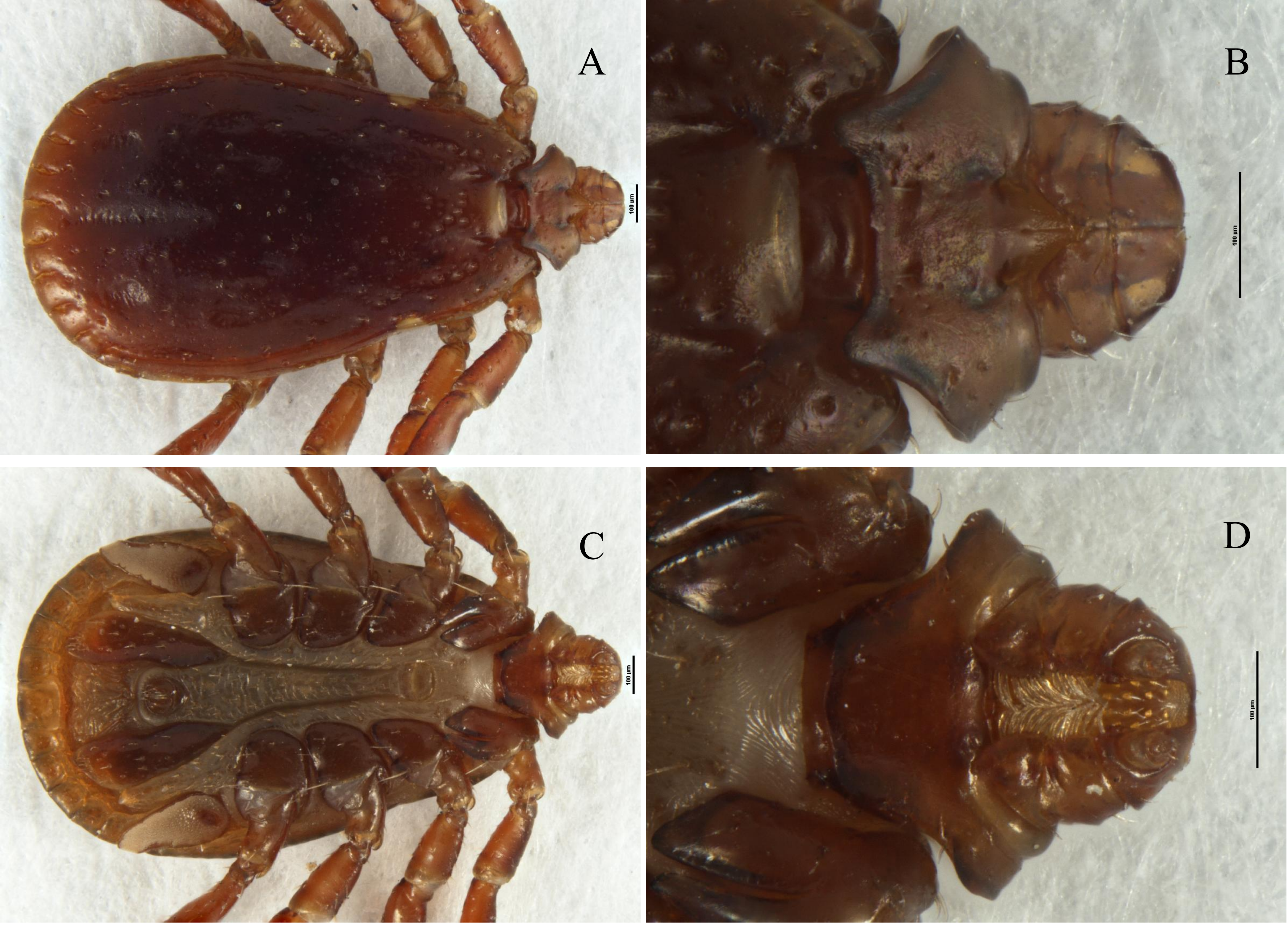

# Ticks from marbled polecat (*Vormela peregusna*)

Figure 10.

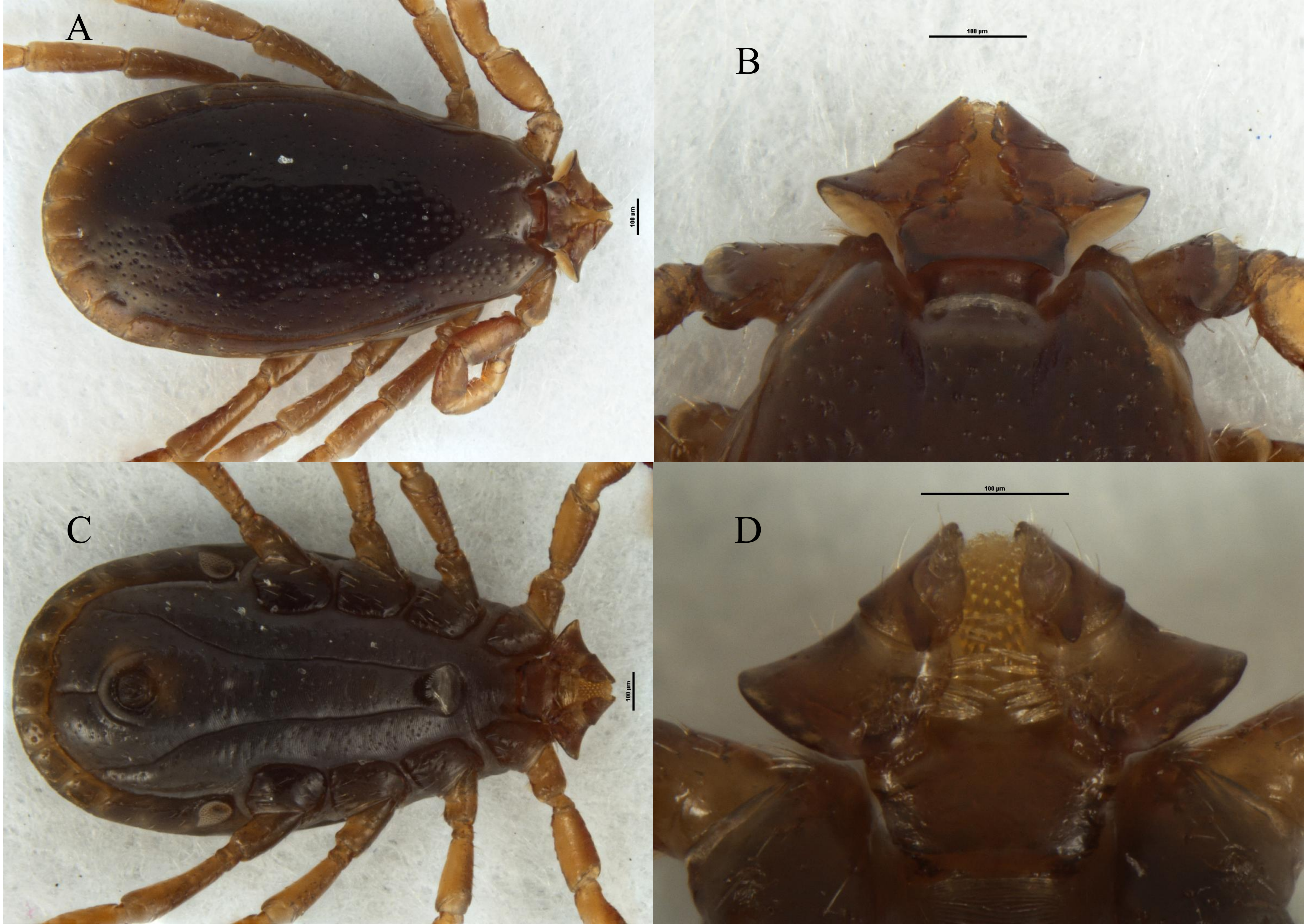

Figure 11.

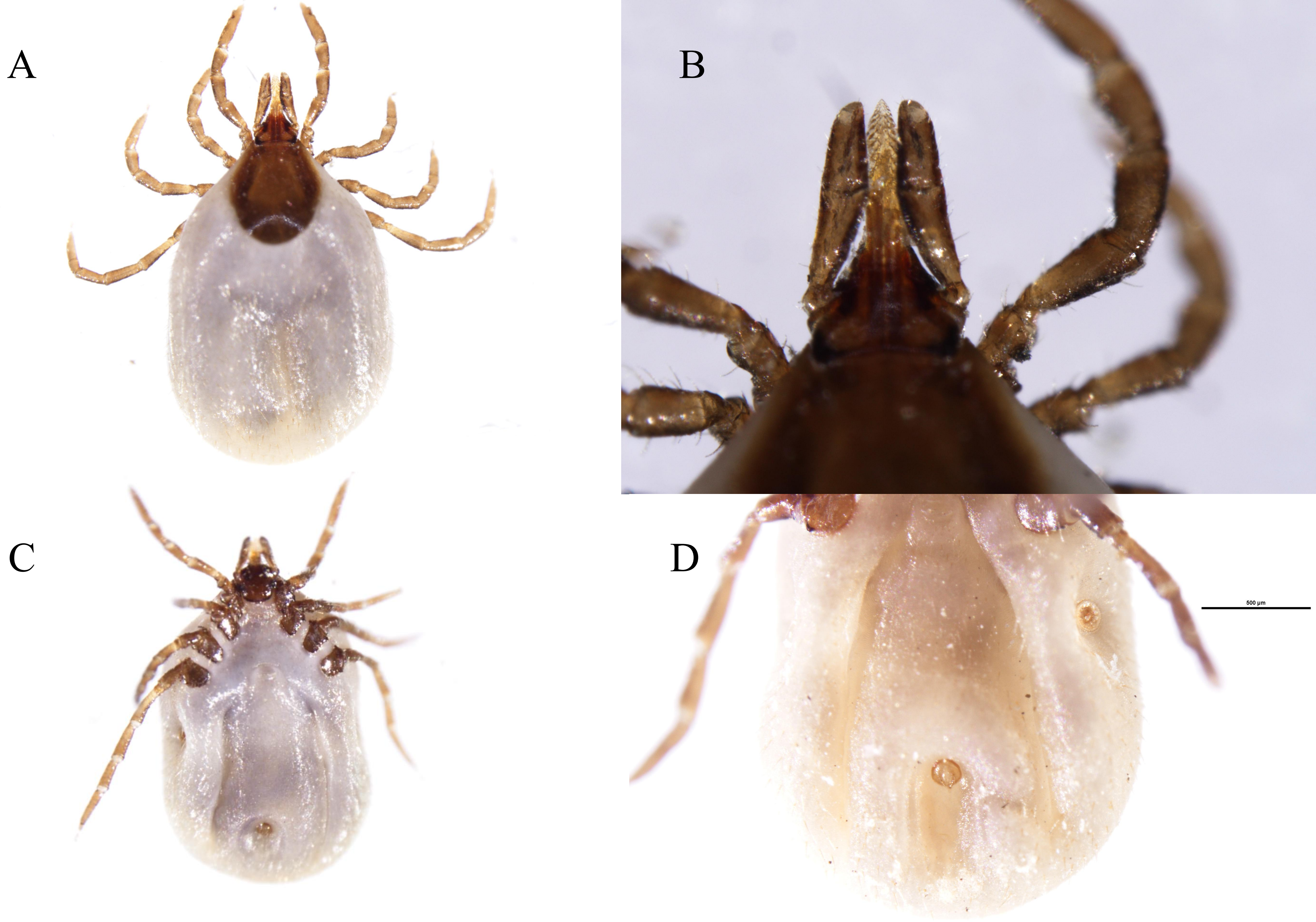

Figure 12.

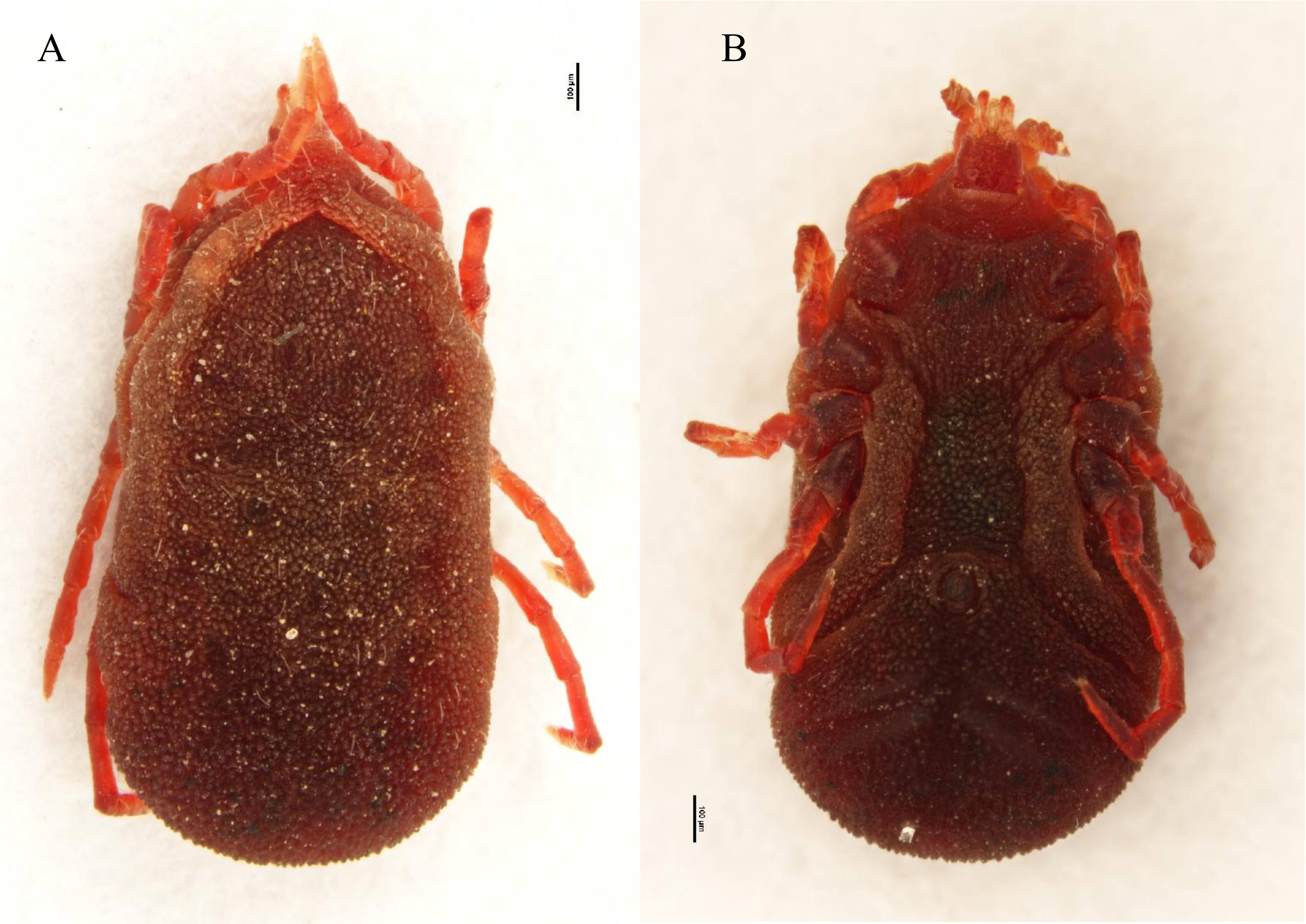

# Ticks from long-eared desert hedgehog (*Hemiechinus auritus*)

Figure 13.

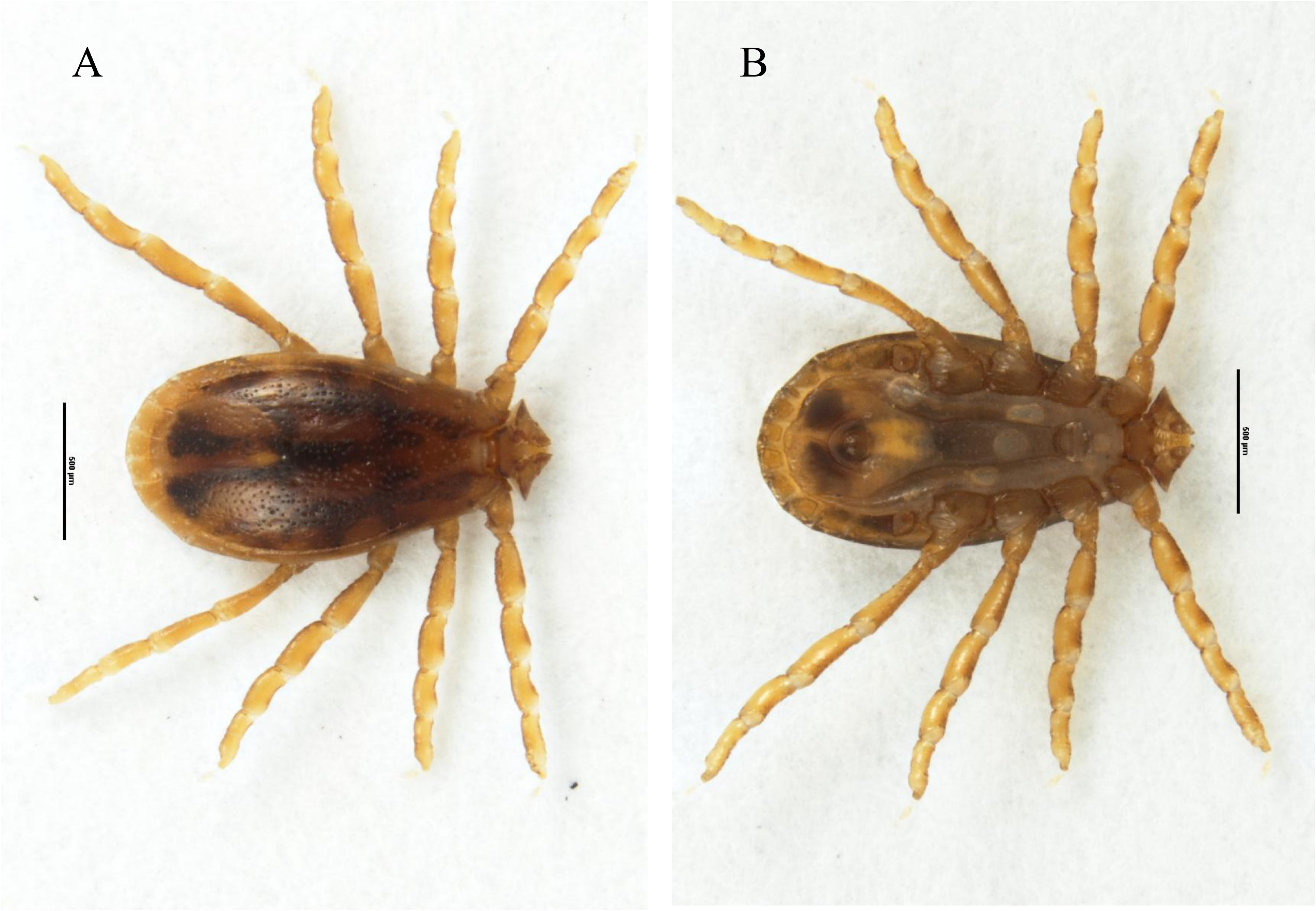

# Ticks from red fox (*Vulpes vulpes*)

Figure 14.

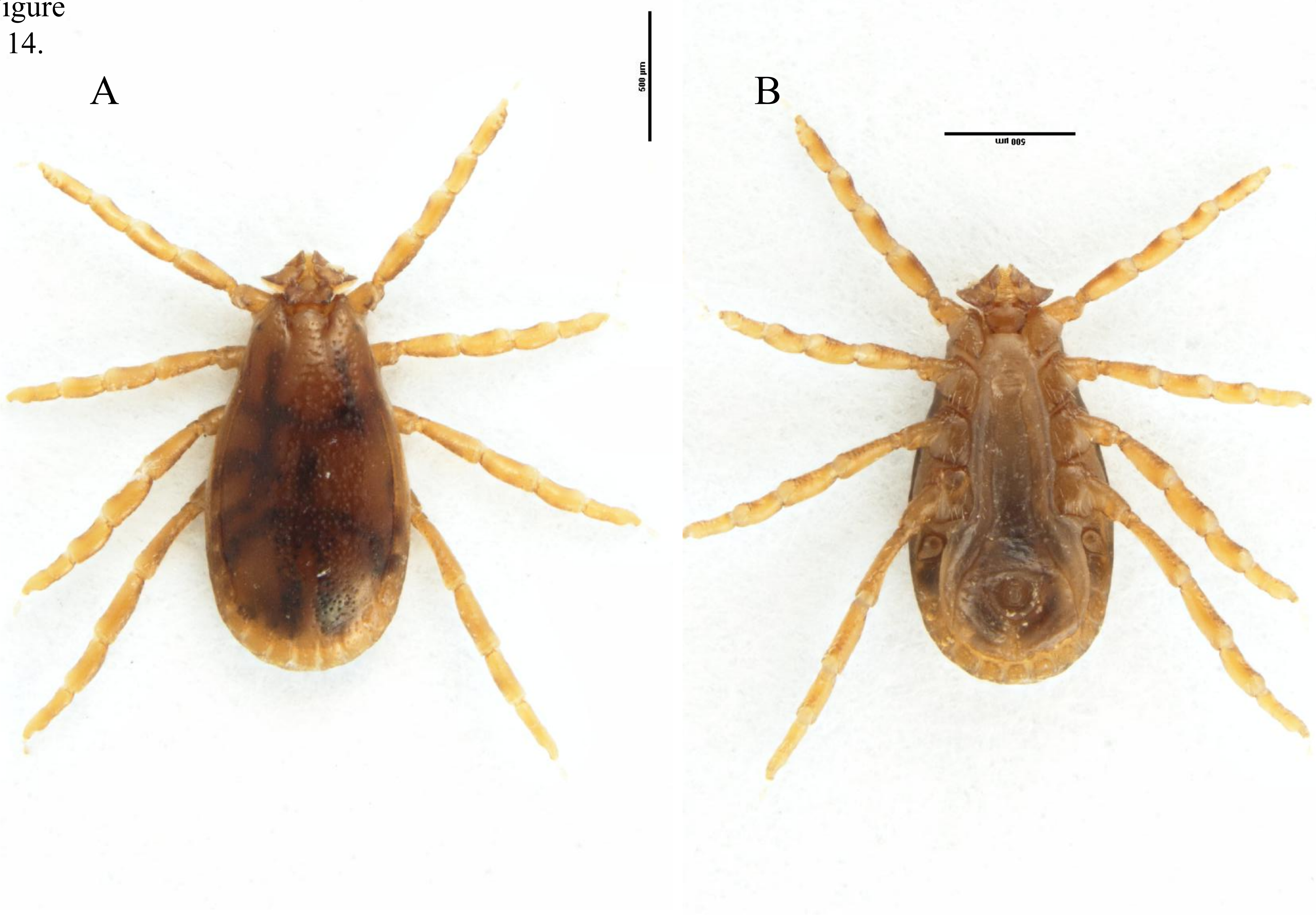

Figure 1.*Hyalomma asiaticum* (larval), A: Dorsal view. B: Ventral view.
Figure 2.*Hyalomma asiaticum* (nymph), A: Dorsal view. B: Basis capituli, dorsal view. C:Ventral view. D: Basis capituli, ventral view.
Figure 3.*Rhipicephalus turanicus* (nymph), A: Dorsal view. B: Ventral view.
Figure 4.*Haemaphysalis erinacei* (larval), A: Dorsal view. B: Ventral view.
Figure 5.*Haemaphysalis erinacei* (nymph), A: Dorsal view. B: Ventral view.
Figure 6.*Ixodes acuminatus* (nymph), A: Ventral view. B: Dorsal view.
Figure 7.*Ornithodoros tartakovskyi* (adult), A: Dorsal view. B: Ventral view.
Figure 8.*Hyalomma asiaticum* (adult), A: Dorsal view. B: Basis capituli, dorsal view. C:Ventral view. D: Basis capituli, ventral view.
Figure 9.*Rhipicephalus turanicus* (adult), A: Dorsal view. B: Basis capituli, dorsal view. C:Ventral view. D: Basis capituli, ventral view.
Figure 10.*Haemaphysalis erinacei* (adult), A: Dorsal view. B: Basis capituli, dorsal view. C:Ventral view. D: Basis capituli, ventral view.
Figure 11. *Ixodes acuminatus* (adult), A: Dorsal view. B: Basis capituli, dorsal view. C: Ventral view. D: Ventral view.
Figure 12.*Ornithodoros tartakovskyi* (adult), A: Dorsal view. B: Ventral view.
Figure 13.*Haemaphysalis erinacei* (adult), A: Dorsal view. B: Ventral view.
Figure 14.*Haemaphysalis erinacei* (adult), A: Dorsal view. B: Ventral view.
