# Supplementary figures and images for: The great gerbil (Rhombomys opimus) as a host for tick species in Gurbantunggut Desert
Source: Parasit Vectors. 2024 Feb 7;17:55. doi: 10.1186/s13071-024-06160-5 (PMC10851595; doi:10.1186/s13071-024-06160-5)

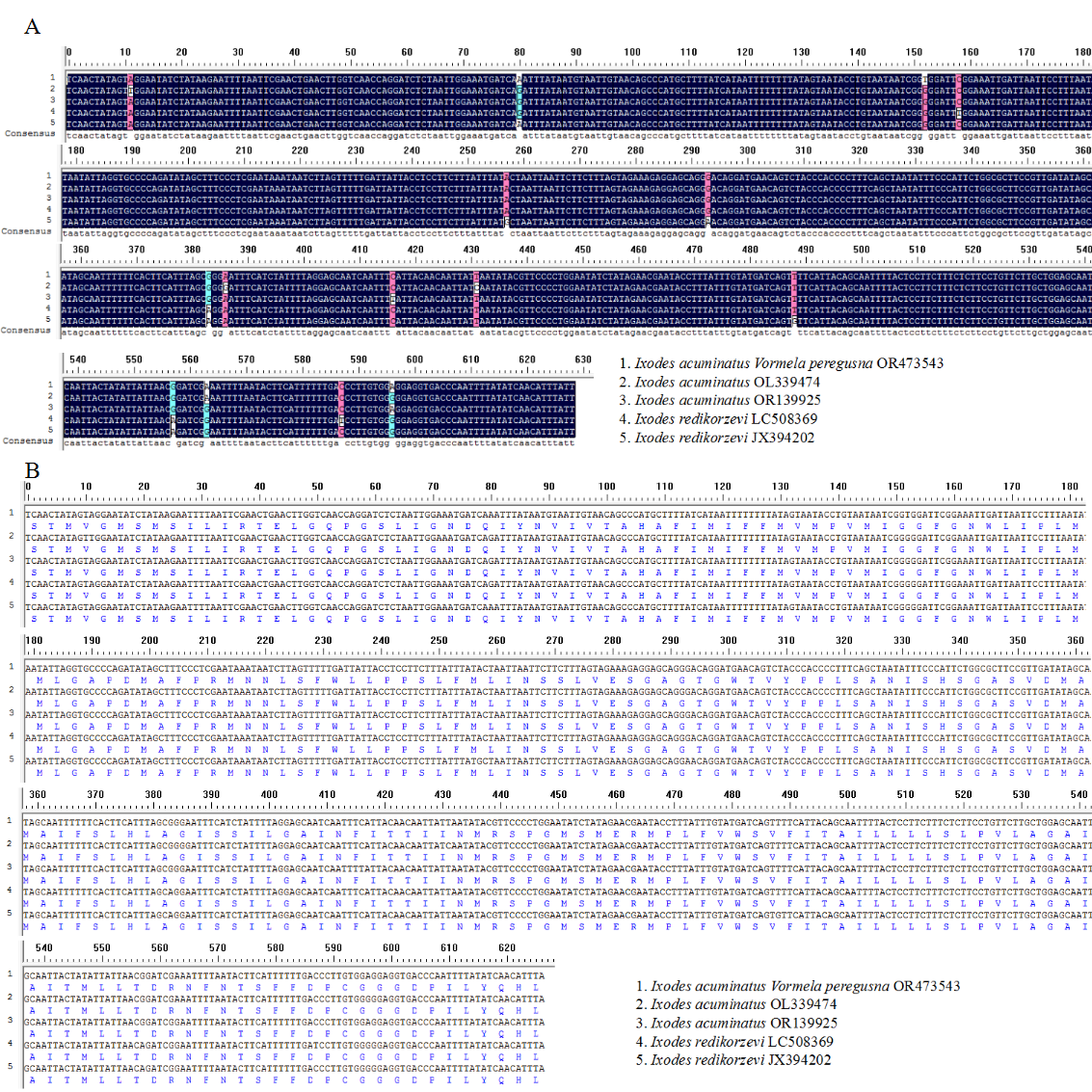

Supplement: Supplementary file 3 — Additional file 3: Figure S2. Nucleic acid (A) and amino acid (B) sequence comparison of Ixodes acuminatus and Ixodes redikorzevi. [file 13071_2024_6160_MOESM3_ESM.tif]

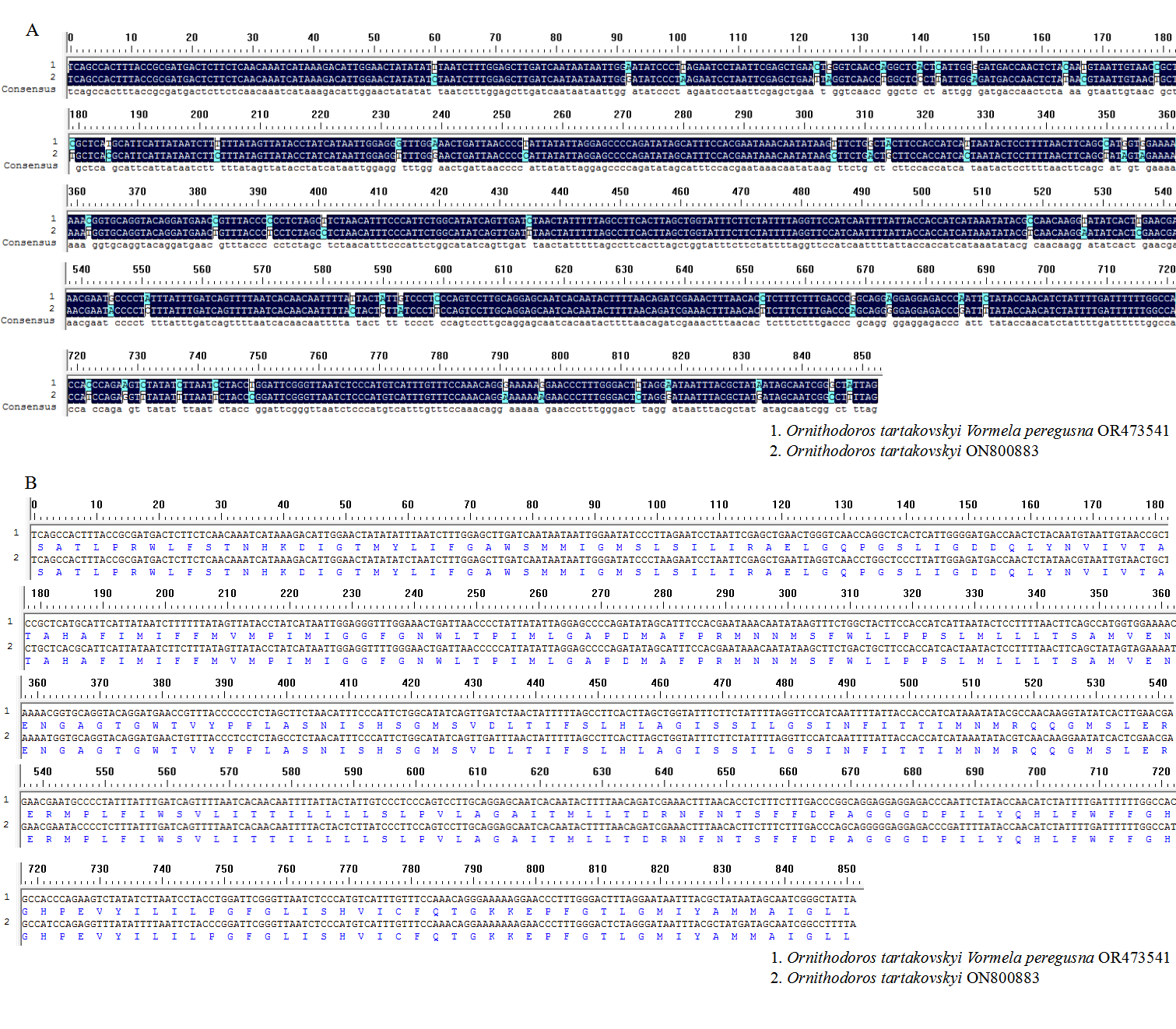

Supplement: Supplementary file 4 — Additional file 4: Figure S3. Nucleic acid (A) and amino acid (B) sequence comparison of Ornithodoros tartakovskyi. [file 13071_2024_6160_MOESM4_ESM.tif]

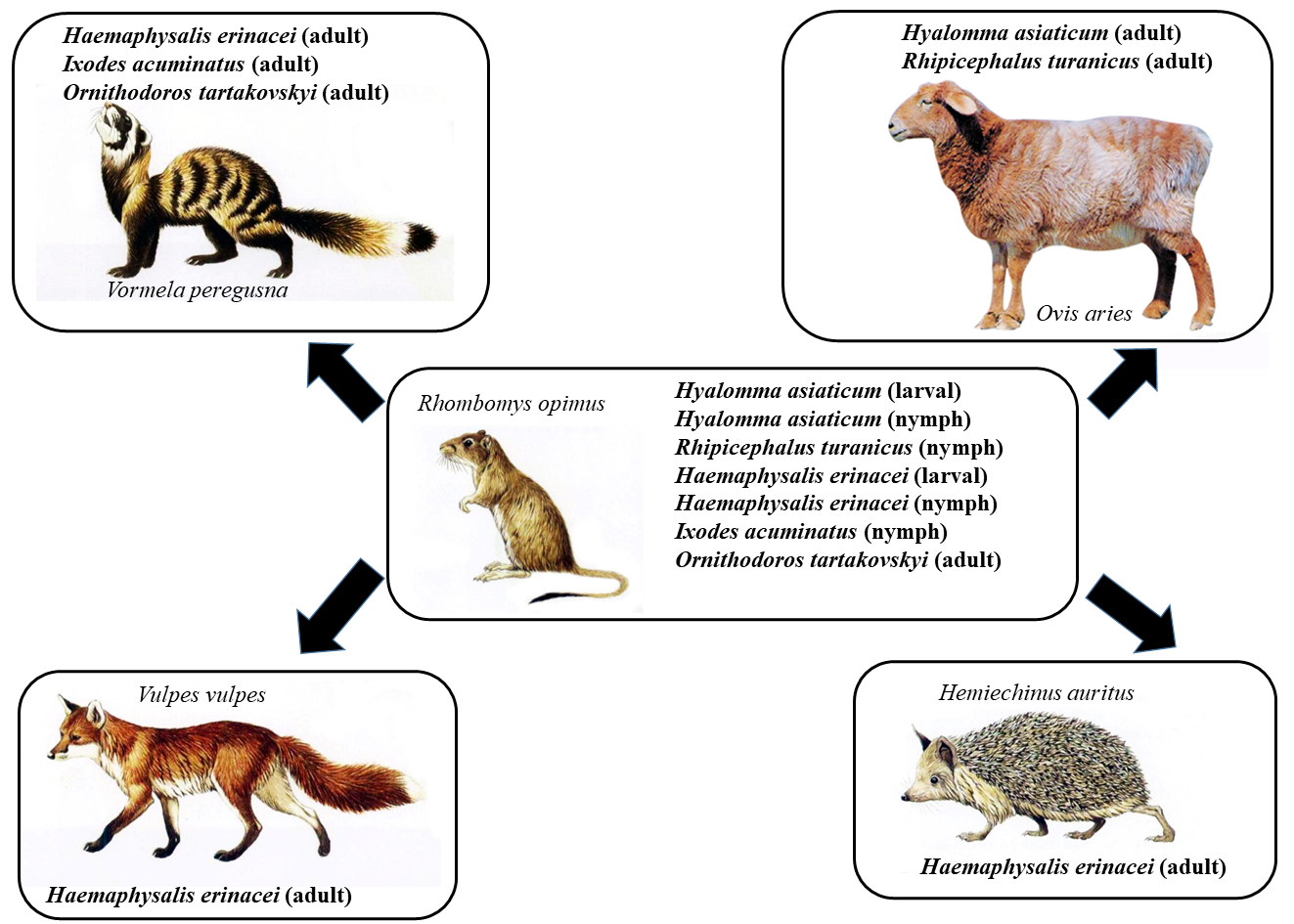

Supplement: Supplementary file 5 — Additional file 5: Figure S4. Schematic diagram illustrating the connectedness of tick hosts in the Gurbantunggut Desert. [file 13071_2024_6160_MOESM5_ESM.tif]
